# Observations on Scarlatina Cynanchica

**Published:** 1808-08

**Authors:** Felix Pascalis


### OBSERVATIONS *on* SCARLATINA CYNANCHICA; *by* FELIX PASCALIS, *M. D.*

WE have been visited during the last winter month by the *Scarlatina Anginosa*, or *Cynanchica* (scarlet sore-throat. This epidemic, I suppose, originates, like all others, in certain specific and deleterious principles of the air, or of our aliment. But whenever it is formed, it is either aggravated by acting and re-acting circumstances, or, if it is counteracted by the predisposing habit of the patient, it unexpectedly proves a very mild complaint. Thus we had two distinct species of the scarlatina cynanchica. Neither prevailed universally; and the worst kind seemed to affect children from the cradle to the age of twelve years. Few adults, except those who were foreigners, have fallen victims to this disease.

Epidemics of rare and distant occurrence are very embarrassing in medical practice. Some time elapses before we have collected all written documents, and recollected our own observations; and if there are but few or confused precepts on their treatment, we cannot place an absolute confidence in our best applications.

In many respects our scarlatina anginosa was similar to that which Fothergill investigated in London, during the years 1747 and 1748, and which, a century before, had pervaded almost all the southern countries of Europe. Withering has still better described the symptoms of the scarlatina, as it prevailed at Birmingham in 1778; so that the disease cannot be mistaken for the malignant or putrid sore-throat, *morbus strangulatorius, suffocativus*, &c. We have likewise a very true description of it by Sauvages, as it raged in Montpellier in 1765. The same I have seen frequently in the most southern parts of France.

Whether this disease is always a concomitant of others, such as the small-pox, measles, and cynanche tonsillaris and trachealis, we have not sufficient authority to affirm: thus much, however, may be said, that it appeared with the measles at Birmingham, and in Philadelphia, where the latter epidemic has not yet subsided, since the beginning of the summer.

Our scarlatina cynanchica was preceded by a few days of stupor and unusual weariness, with a slight painful sensation in the throat, and sometimes in the neck and back part of the head. Children who could speak complained of an alternating chilliness and flushing heat, head-ach,

 and and sickness. In this stage of the disease, many of the worst cases were marked with delirium, convulsive motions of the muscles of the face, and grinding of the teeth. On the second and third day the soreness of the throat had so much increased that deglutition was very painful; yet the tonsils and salivary glands were little enlarged; but the tongue, velum palati, and fauces, were uncommonly red. Already the face and neck appeared flushed; and, from the breast down to the pubes, a florid redness seemed to be produced by innumerable small pimples and pustules. This eruption sometimes extended to the lower extremities, and over the shoulders and the hips.

Convulsions and delirium in some cases, and great restlessness in others, were observed; and every symptom changed according to the impending issue of the case. If fatal, on the fourth day the scarlet eruption abated, the belly swelled, the skin became rough and dry, and the redness of the cheeks and lips gave place to a livid paleness of the face. The patient experienced an uncommon thirst, without ever swallowing but a few drops; his mouth became parched, and his pulse was small, quick and feeble. The breath was offensive, respiration tremulous and accelerated; and at last, any kind of medicine would induce a colliquative diarrhœa. In a few hours death ensued, marking the surface of the body with a dark brown ecchymosis. But on the fourth day the case might take a far different and more favourable aspect: the scarlet eruption might change into a brownish colour, some moisture be perceived on the skin, and the submaxillary glands become more tumefied. Although the pain of the throat and the difficulty of deglutition were augmented, and the tongue seemed covered with a black or yellow crust, still a favourable discharge of purulent matter was effected from the nose and mouth. Branny scales were discovered on every part of the body, and gangrenous sloughs were discharged from the fauces.

I have not seen, in this stage, the same external effects of mortification, or of hæmorrhage from the ears, &c. as many writers relate; but I believe parts were frequently spotted, and a few gangrenous sloughs detached from them. Notwithstanding, the bowels were regular, and obedient to operations; the urine appeared natural, and the pulse equal, although quick and febrile. In short, in this stage, the scarlatina cynanchica existed no more, except by its effects; the case was merely a typhoid disease, with more or less of those accidents, which were to be referred either either to the identity of the attack, or to the age of the patient.

Dissection has, as yet, afforded but little elucidation as to the nature of this disease. Withering mentions one only of Sennertus, who found great ravages in the lungs and the liver. But these, I have reason to believe, cannot be ascribed to the scarlet fever; they must have been the result of some previous complaint. A boy of eleven years old was, on the fourth day, carried off, with the most severe symptoms of the epidemic, Permission of opening the body having been obtained, no alteration was discovered in the lungs nor in the liver. The trachea and the stomach were likewise in a very sound state. The fauces, it is true, the glottis and tonsils, were remarkably livid. But, on the other hand, the large intestines were mostly mortified, and all the glands of the mesentery were considerably enlarged and indurated. In this case also I am prevented from drawing any positive conclusion, for the child was supposed to have been previously labouring under some consumptive disorder.

I have heard many eminent physicians describe two very different degrees of the scarlatina cynanchica. Thus, while natural exertions will be sufficient for the management of a mild case, we may be at a loss to fix upon the best mode of treating gangrenous scarlatina. Medical writers have rather increased our perplexity on this subject, by informing us that certain remedies have either failed or succeeded, when they have neglected to form indications to rule the practice and exhibition of medicines. The prolix treatise of Withering determines nothing but a preference in favour of emetics and diuretics. Can it be conceived, however, that, in an inflammatory and malignant disease, any remedy, such as bleeding, purging, sweating, tonics, acids, opium, blisters and bathing, could all and severally prove abortive, or contrary to necessary indications?

Judging from our experience with Dr. Withering's favourite resource of emetics, we find that they answer very well in the beginning of the disease, to discharge the contents of the stomach, to dispel the impending influx of fluids in the fauces, and to keep up the scarlet eruption on the skin until its desquamation is produced. But this first period of the disease is short; and the more intense it is, the greater is the danger of promoting, even with mild medicines, an inverted operation, when a fatal diarrhœa rhœa will soon put an end to the struggle and the life—*quæque miserrima vidi.*

As for his diuretic (fixed vegetable alkali), it afforded relief if given as a *citrate of pot-ash.* It promoted salutary discharges, but its operation did not concur with the principal indications of the treatment; and hence I may show the efficacy of many of the remedies rejected by, or unknown to Dr. Withering.

Three things are striking when the scarlet cynanche has favourably passed the periods of invasion and *maturation.* These are, 1st. The efflorescence or desquamation on the skin; 2dly. The suppuration or discharges of the mouth, nose and ears; 3dly. The gangrenous sloughs from the fauces, or the gangrenous spots, abscesses, vibices, &c. on any part of the body. Of whatever nature the poison or virus of the disease may be, it is surely requisite to aid its dispersion in the most natural manner; viz. the scarlet desquamation should be carefully promoted; the suppuration in the fauces should be accelerated; and the gangrenous tendency in the system should always be counteracted.

In the first instance one or two emetic and diaphoretic remedies will prove highly beneficial; but how much more effectually the second end will be answered by calomel, is evinced by the happy concurrence of many facts. A dose of four or five grains, with an equal quantity of camphor, mixed with some jelly, may be repeated every three or four hours, and seldom continued longer than three or four days, when the purulent discharges from the mouth will be sufficiently promoted, and when bark, wine, opium, and even alcoholic mixtures, will effectually oppose the gangrenous tendency.

If we attend to several circumstances, throughout the different stages of the disease, it will appear obvious that its invasion and action must accumulate such excitement, that bleeding and warm bathing ought sometimes to be resorted to for the relief of convulsive and inflammatory symptoms. But, after the desquamation and discharges of the mouth have taken place, as the disease is transformed into a typhoid affection, in which a direct debility is induced, it will demand the use of all stimulating remedies, among which blisters are particularly successful.

The exhibition of mercury in scarlatina cynanchica, assisted by a medical process, according to the above-mentioned intentions, has never been unsuccessful, as far I employed it, in a few cases of the most intense malignancy.

nancy. Yet much benefit is to be ascribed to calomel, according to the recommendation long ago given to it by Dr. Samuel Bard, of New-York, in his Essay on the sore-throat, or *Morbus Suffocativus*,* and which, perhaps, differed but little from our epidemic. Had I no other authority, I must acknowledge that I should have been sufficiently encouraged by Dr. Rush, in one of those remarkable cases in which the organical powers were so much prostrated and injured that life was despaired of.

*P. S.* In the Medical Repository, vol. v. p. 39, a case is related of the tape-worm being effectually discharged by using the decoction of Fern.

Is not this plant the *Polypodium Filix Mas* of Linn. Class *Cryptogamia*, called *La Fougere* by the French?

Does not its pulverized root constitute the famous remedy of Mrs. Nouffer of Switzerland, against the tænia or tape-worm?

Did not Lewis XVI. King of France, order the remedy to be bought, and published, as it is, in many books, and especially the Pharmacy of Beaumé?

Was the gentleman who used it unacquainted with this form of the remedy before he prescribed the decoction, or did he not rely upon the method of exhibiting the root itself in powder? If so, I take the liberty to inform him that I was once present at the operation of two drachms of the said powder, which, in twenty minutes, expelled from the body of a Russian Captain as many feet of a tape-worm as to fill up a quart bottle!

*Philadelphia, Feb,* 8. 1802.

---

### *Memoir upon the Muriatic Ether, lately read at the Institute of France.* By M. Thenard.†

AFTER having examined why the muriatic either has hitherto remained almost unknown among chemists, although frequently the object of chemical experiments, the author gives the method of obtaining it. For this purpose, as the above ether is habitually in the state of gas, the following apparatus must be employed.

We

---

* Vide American Philosophical Transactions, vol. i.

